# Gut microbiota transplantation from db/db mice induces diabetes-like phenotypes and alterations in Hippo signaling in pseudo germ-free mice

**DOI:** 10.18632/aging.104101

**Published:** 2020-11-20

**Authors:** Fan Yu, Riyue Jiang, Wei Han, Gaofeng Zhan, Xiaolin Xu, Xiaohong Jiang, Long Wang, Shoukui Xiang, Qin Zhou, Cunming Liu, Bin Zhu, Fei Hua, Chun Yang

**Affiliations:** 1 Department of Endocrinology, The Third Affiliated Hospital of Soochow University, Changzhou 213003, China; 2 Department of Ultrasound Imaging, Renmin Hospital of Wuhan University, Wuhan 430060, China; 3 Department of Neurosurgery, The Third Affiliated Hospital of Soochow University, Changzhou 213003, China; 4 Department of Anesthesiology, Tongji Hospital, Tongji Medical College, Huazhong University of Science and Technology, Wuhan 430030, China; 5 Department of Anesthesiology, The First Affiliated Hospital of Nanjing Medical University, Nanjing 210029, China; 6 Department of Critical Care Medicine, The Third Affiliated Hospital of Soochow University, Changzhou 213003, China

**Keywords:** gut microbiota, type 2 diabetes mellitus, Hippo signaling, pseudo germ-free mice

## Abstract

Type 2 diabetes mellitus (T2DM) is an age-related metabolic disease that is of increasing concern. Gut microbiota might have a critical role in the pathogenesis of T2DM. Additionally, Hippo signaling has been associated strongly with the progression of T2DM and the aging process. We adopted db/db male mice as a T2DM model, and the gut microbiota of db/db and m/m mice were transplanted successfully into pseudo germ-free mice. Furthermore, Hippo signaling, including mammalian sterile 20-like protein kinases 1 (MST1), large tumor suppressors 1 (LATS1), Yes-associated protein (YAP), and phosphorylation of YAP (p-YAP) in peripheral tissues were significantly altered and highly correlated with blood glucose in db/db mice. Interestingly, the host after gut microbiota transplantation from db/db mice showed decreased MST1 and LATS1 levels, and p-YAP/YAP ratio in the heart, liver, and kidney compared to those from m/m mice. Negative correlations between fasting blood glucose and Hippo signaling levels in selected peripheral tissues also were identified. These findings suggest that alterations in Hippo signaling in selected peripheral tissues may contribute to the development of T2DM, and that therapeutic interventions improving Hippo signaling by gut microbiota transplantation might be beneficial for the treatment of T2DM and other age-related metabolic diseases.

## INTRODUCTION

Worldwide, type 2 diabetes mellitus (T2DM) in individuals older than 65 years is gradually becoming a prevalent public concern [1, 2] because of its severe disability and mortality in the aging population [3]. Additionally, increasing age aggravates the risk of impaired fasting glycemia associated with glucose intolerance level, which potentially contributes to the onset of T2DM [4–6]. In this regard, T2DM is an age-related metabolic disorder involving severe complications, especially cardiovascular and neurodegenerative diseases occurring in the elderly [7, 8].

We previously reported that abnormal composition of gut microbiota greatly contributed to cognitive decline in streptozotocin-induced diabetic mice and Alzheimer’s disease’s (AD) mouse model-senescence-accelerated mouse prone 8 mice [9, 10]. Although the role of gut microbiota in age-related diseases has not yet been elucidated, evidence has shown that transplantation of gut microbiota from healthy donors effectively improved age-related neurodegenerative disorders, such as AD [11] and Parkinson’s disease [12, 13]. Furthermore, a new concept has been proposed that the gut–brain axis is responsible for the complex relationship between gut microbiota and the central nervous system [14–16]. It is therefore that gut microbiota might be highly related with neurodegenerative diseases attributing to its environmental role in energy metabolism.

There also is a causal linkage between T2DM characterized by insulin resistance and obesity in pathology and gut microbiota dysbiosis [17–19]. According to our previous study, alterations in gut microbiota composition contributed to T2DM development in db/db mice, and transplantation of gut microbiota could alleviate the metabolic parameters, consisting of fasting glycemia, body weight, food and water intake [20]. The metabolic parameters of a systemic body appear to be on a downward trend with increasing age due to the decline in metabolic rates [21, 22]. Accordingly, dysregulation of metabolism with increasing age eventually becomes a predisposing factor for T2DM [7], combined with the abnormal composition and function of gut microbiota.

Hippo signaling is recognized as a key regulator of organ size and tissue homeostasis [23, 24]. It comprises mammalian sterile 20-like protein kinases 1 and 2 (MST1/2), large tumor suppressors 1 and 2 (LATS1/2), Yes-associated protein (YAP), and transcriptional coactivator with PDZ-binding motif [25, 26]. Furthermore, Hippo signaling also has a pivotal role in modulation of cellular proliferation and apoptosis that can in turn regulate metabolic homeostasis [27]. A clinical study enrolling nine healthy subjects and nine patients with T2DM demonstrated 778 differentially expressed genes in the livers, and Hippo signaling was a key pathway in the progression of T2DM [28]. In our previous study, aberrant expressions of Hippo signaling were detected in selected cerebral and peripheral tissues in streptozotocin-induced diabetic mice accompanied by cognitive dysfunction [29]. Hippo signaling also is involved in the mechanisms underlying the aging process, under the interaction with other signaling pathways, such as AMP-activated protein kinase and the sirtuin pathways [30]. Hence, a study of the relationship between Hippo signaling and age-related metabolic disorders, such as T2DM, is urgently needed.

Although recent work has uncovered novel mechanisms of mitochondrial dysfunction in peripheral tissues implicated in T2DM progression [31], the role of peripheral organs in T2DM development remains ambiguous. Considering the critical role of liver and muscle tissues in energy synthesis and metabolism [32, 33], combined with the close relationship between gut microbiota and T2DM, we identified expressions of Hippo signaling in selected peripheral tissues, including heart, liver, kidney, muscle, and gut in db/db mice and pseudo germ-free mice after gut microbiota transplantation. Furthermore, correlation analyses were carried out between fasting glycemia and expressions of Hippo signaling in selected peripheral tissues to verify the causal linkage.

## RESULTS

### Comparisons of metabolic parameters between db/db and m/m mice

**Figure 1:**
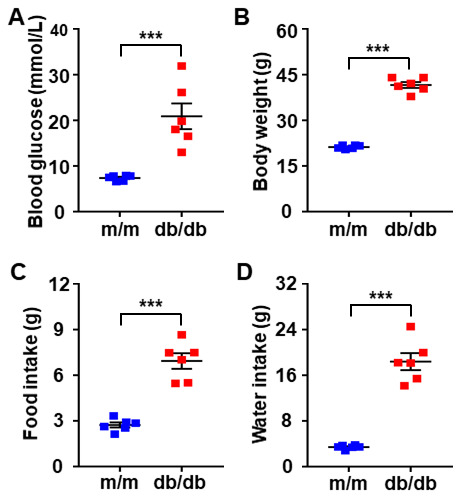
**Comparisons of metabolic parameters in db/db and m/m mice.** (**A**) Blood glucose. (**B**) Body weight. (**C**) Food intake. (**D**) Water intake. Data are shown as mean ± SEM (*n* = 6). *P < 0.05, **P < 0.01, or ***P < 0.001. N.S., not significant.

We adopted db/db mice as a T2DM mouse model and m/m mice as control subjects. Metabolic parameters, including blood glucose, body weight, and food and water intake, between the db/db and m/m mice were evaluated after 1 week of acclimation. Fasting blood glucose, body weight as well as food and fluid intake were significantly increased in db/db mice than those of m/m mice (Figure 1A–1D).

### Expressions of Hippo signaling in selected peripheral tissues between db/db and m/m mice

**Figure 2:**
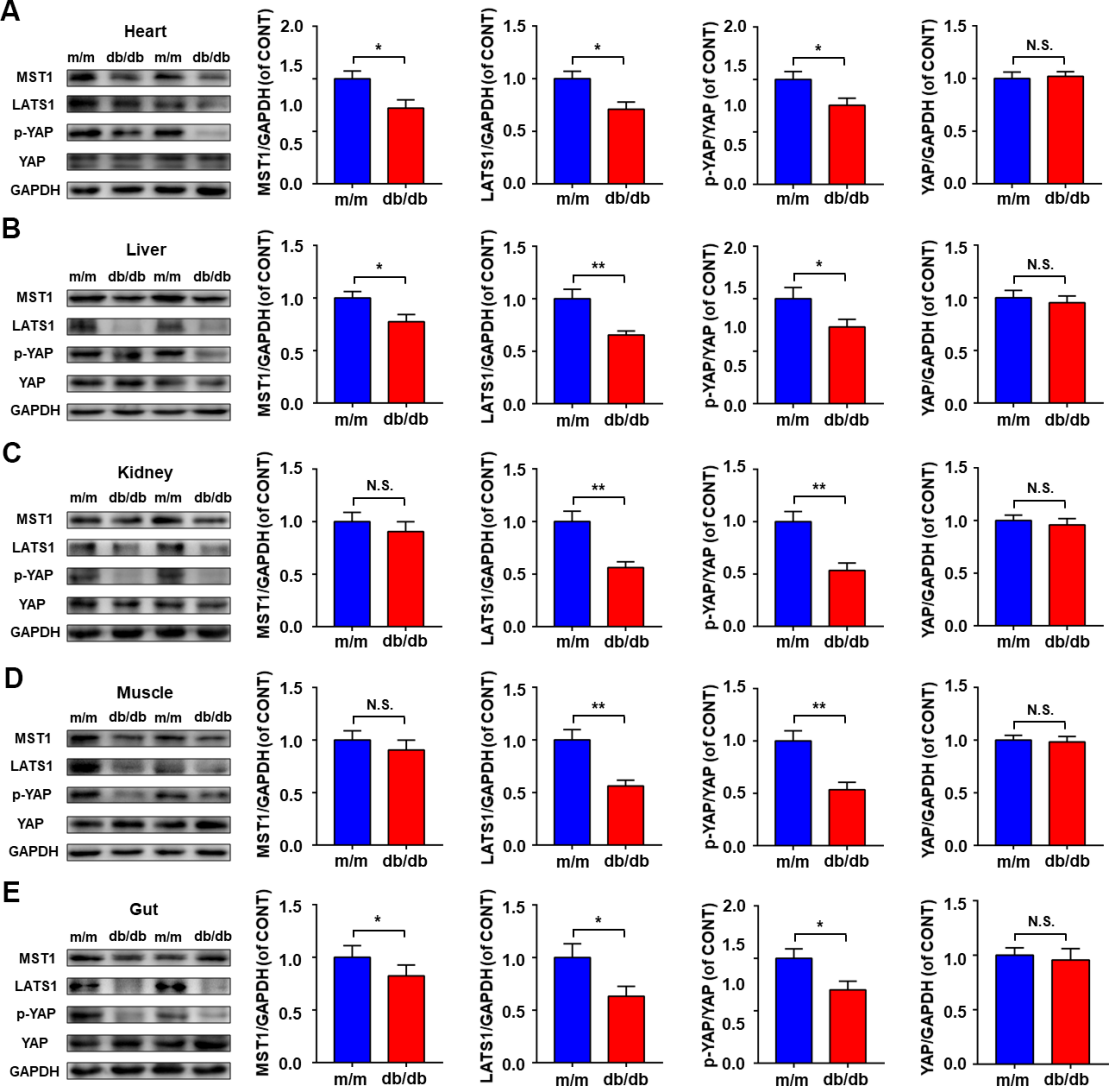
**Hippo signaling levels in peripheral tissues between db/db and m/m mice.** MST1, LATS1, p-YAP/YAP ratio, and YAP in the heart (**A**), liver (**B**), kidney (**C**), muscle (**D**), and gut (**E**). Data are shown as mean ± SEM (*n* = 6). *P < 0.05, **P < 0.01, or ***P < 0.001.

Expressions of Hippo signaling, consisting of MST1, LATS1, p-YAP, and YAP were determined in selected peripheral tissues between db/db and m/m mice (Figure 2A–2E). Compared to m/m mice, db/db mice showed a significant decrease in MST1, LATS1, and p-YAP/YAP ratio in the heart, liver, and gut (Figure 2A, 2B, 2E). Although the MST1 level in kidney and muscle tissues failed to show a significant change between db/db and m/m mice, the LATS1 levels and p-YAP/YAP ratio in db/db mice were significantly lower than those in m/m mice (Figure 2C, 2D).

### Correlations between fasting blood glucose and Hippo signaling levels in selected peripheral tissues between db/db mice and m/m mice

**Figure 3:**
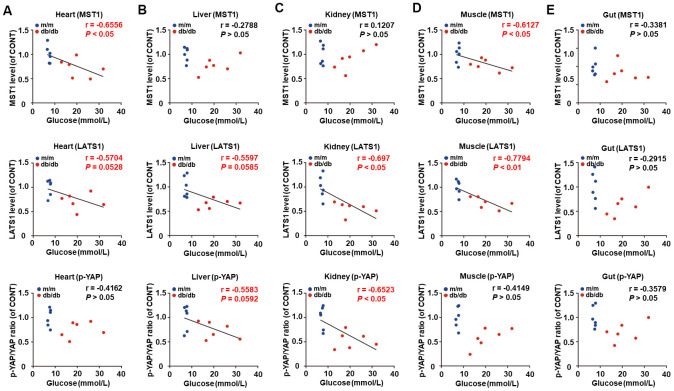
**Correlations between fasting blood glucose and Hippo signaling levels in peripheral tissues between db/db mice and m/m mice (*n* = 12).** MST1, LATS1, and p-YAP/YAP ratio in the heart (**A**), liver (**B**), kidney (**C**), muscle (**D**), and gut (**E**).

Considering the pivotal role of fasting blood glucose in the progression of T2DM, correlations between fasting blood glucose and Hippo signaling levels were analyzed (Figure 3A–3E). Intriguingly, there were significant negative correlations between fasting blood glucose and MST1 and LATS1 levels in the heart and muscle (Figure 3A, 3D), as well as LATS1 levels and p-YAP/YAP ratio in the liver and kidney (Figure 3B, 3C). However, there was no significant correlation between fasting blood glucose and Hippo signaling levels in the gut (Figure 3E).

### Impacts on metabolic parameters by fecal transplantation into pseudo germ-free mice

**Figure 4:**
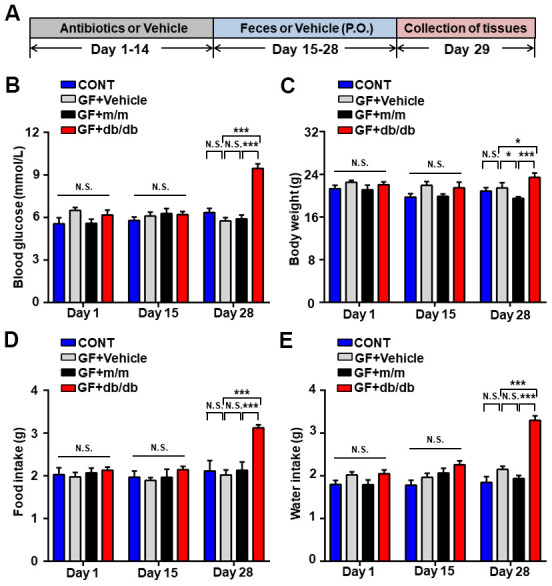
**Alterations in the metabolic parameters after fecal transplantation in pseudo germ-free mice.** (**A**) Schedule of our study. Mice were treated orally with fecal microbiota from db/db or m/m mice for 14 consecutive days after receiving large doses of antibiotics for 2 weeks. Peripheral tissues were collected for subsequent experiments on day 29. (**B**) Blood glucose. (**C**) Body weight. (**D**) Food intake. (**E**) Water intake. Data are shown as mean ± SEM values (*n* = 7). *P < 0.05, **P < 0.01, or ***P < 0.001. CONT, control; GF, germ-free.

After the establishment of pseudo germ-free mice, fecal transplantation were conducted from db/db and m/m mice (Figure 4A). Then, metabolic parameters were measured on day 28 as reported previously [20]. Obviously, as compared to m/m or vehicle group, fasting glycemia, body weight, food and fluid intake were significantly elevated in db/db group. However, there were no dramatic alterations among four groups on day 1 and 15 (Figure 4B–4E).

### Hippo signaling levels in selected peripheral tissue after fecal transplantation in pseudo germ-free mice

**Figure 5:**
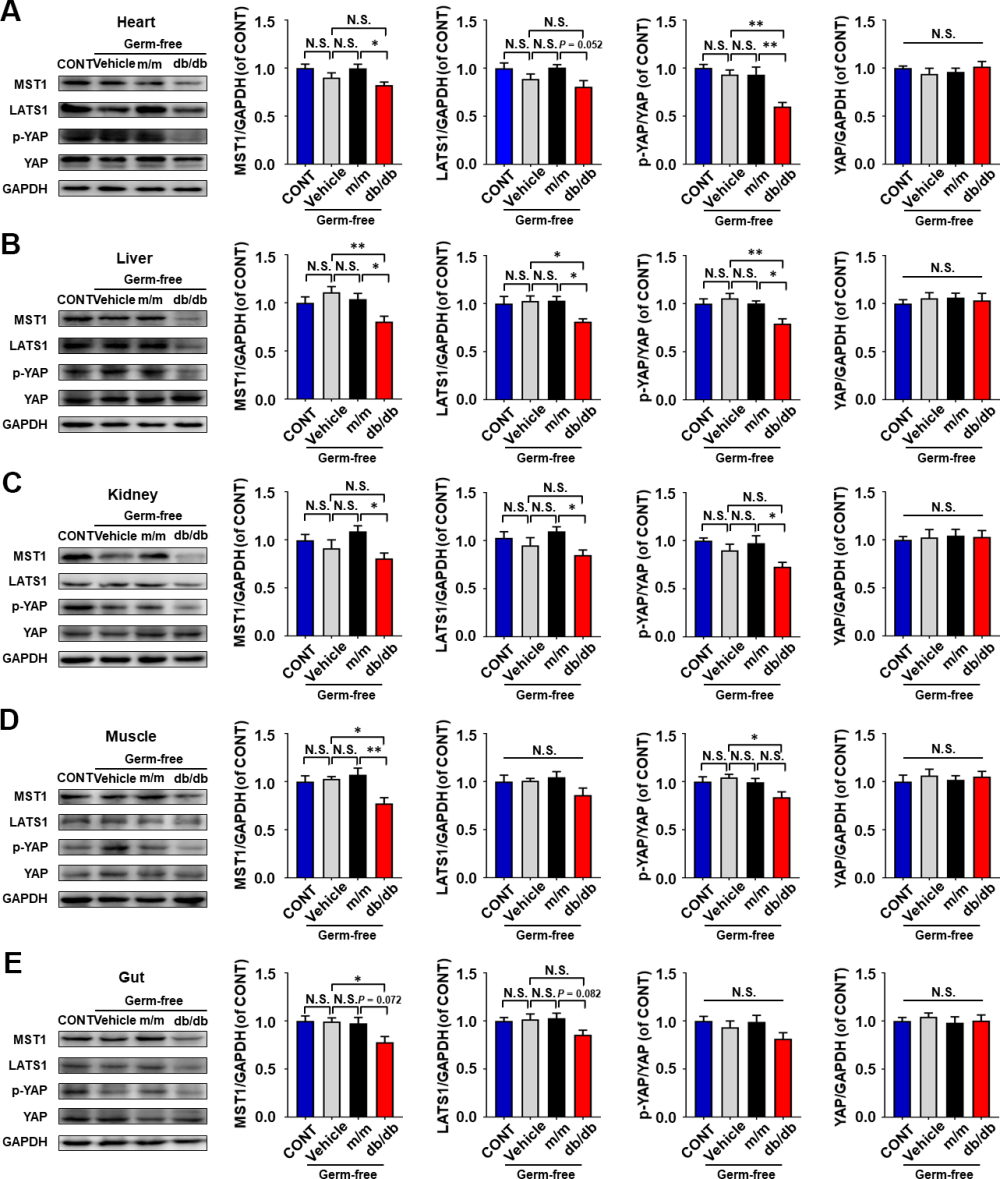
**Hippo signaling levels in peripheral tissues after fecal transplantation among pseudo germ-free mice.** (**A**) MST1, p-YAP/YAP ratio, and YAP in the heart. Also shown are MST, LATS1, p-YAP/YAP ratio, and YAP in the liver (**B**), kidney (**C**), muscle. (**D**), and gut (**E**). Data are shown as mean ± SEM (*n* = 7). *P < 0.05, **P < 0.01, or ***P < 0.001. CONT, control.

After fecal transplantation, MST1 and LATS1 levels, and p-YAP/YAP ratio in selected peripheral tissues were determined by Western blot in pseudo germ-free mice (Figure 5A–5E). Intriguingly, a significant decrease in these values was observed in the liver tissue of db/db mice compared with values in the vehicle or m/m group (Figure 5B). MST1 levels and p-YAP/YAP ratio were significantly decreased, and there was a slight decrease in LATS1 level in the hearts of the db/db group compared to values in the m/m group (Figure 5A). Regarding kidney tissue, a significant decrease was identified in the MST1 and LATS1 levels, and p-YAP/YAP ratio in the db/db group compared to those in m/m group (Figure 5C). Additionally, the MST1 level and p-YAP/YAP ratio in muscle of the db/db group showed a significant decrease compared to those in the vehicle group, whereas no significant difference was detected in LATS1 level among the four groups (Figure 5D). Although the MST1 level was significantly lower in the gut of the db/db than in the vehicle groups, there was no statistical difference in MST1 and LATS1 levels, and p-YAP/YAP ratio between the db/db and m/m groups (Figure 5E). However, Hippo signaling expression in all selected peripheral tissues failed to show a significant alteration among the control, vehicle, and m/m groups (Figure 5A–5E).

### Correlations between fasting blood glucose and Hippo signaling levels in selected peripheral tissues after fecal transplantation in pseudo germ-free mice

**Figure 6:**
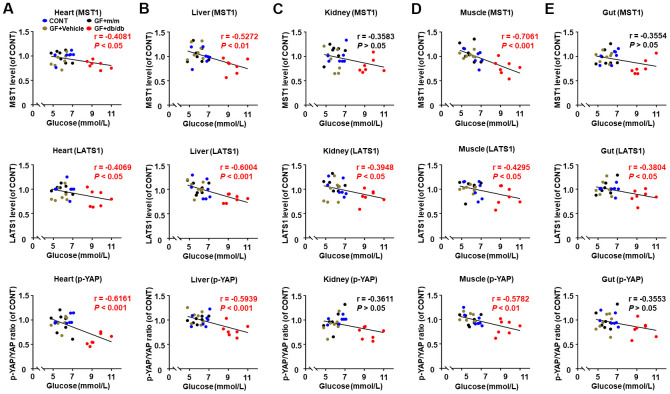
**Correlations between fasting blood glucose and Hippo signaling levels in peripheral tissues after fecal transplantation in pseudo germ-free mice (*n* = 28).** MST1, LATS1 and p-YAP/YAP ratio in the heart (**A**), liver (**B**), kidney (**C**), muscle (**D**), and gut (**E**).

A significant correlation was noted between fasting blood glucose and MST1, LATS1 levels, and p-YAP/YAP ratio in the heart, liver, and muscle tissues, as analyzed in selected peripheral tissues after fecal transplantation from db/db and m/m mice into pseudo germ-free mice (Figure 6A, 6B, 6D). A significant negative correlation was found between fasting blood glucose and LATS1 levels in the kidney and gut. However, there were no significant correlations between fasting blood glucose and MST1 level and p-YAP/YAP ratio (Figure 6C, 6E).

## DISCUSSION

It is generally acknowledged that aging plays a predisposing role in the dysregulation of glucose metabolism [34]. Age-related insulin- sensitivity decline, accompanied by hyperglycemia, might disequilibrate glucose homeostasis, possibly triggering a potential onset of T2DM [35]. It is notable that, the prevalence of T2DM in the aged patients especially those over 65-year-old is rising more than 25% [36, 37]. In this regard, T2DM is recognized as an age-related metabolic disease. In this study, C57 BL/KS db/db male mice were adopted as a T2DM rodent model to further investigate the mechanisms involved in T2DM progression. Although various animal models have been adopted in the study of T2DM, db/db mice are confirmed as a genetically diabetic rodent model because of the missense mutations of leptin receptors [38, 39], pathologically characterized by leptin resistance, namely imbalance between higher circulatory levels of leptin and lower leptin receptors [40]. Consequently, the levels of fasting glycemia, body weight, food and fluid intake were significantly elevated in db/db mice than m/m mice, which are in agreement with the clinical features in T2DM patients [41].

In fact of growing incidence of T2DM in elderly populations, more attentions have focused on the intricate pathogenesis of T2DM that mainly attributed to genetic susceptibility and environmental risk factors [42, 43]. Recently, multiple lines of studies have revealed that gut microbiota, a pivotal role in environmental factors, essentially contributed to the development of metabolic disorders, as well as age-related neurologic disorders [44]. Despite the obscure mechanism of gut microbiota in age-related diseases, several lines of evidence suggested that gut microbiota transplanted from healthy donors can effectively alleviate T2DM progression [45] and age-related neurodegenerative disorders, including AD and Parkinson’s disease [11, 13]. In our study, pseudo germ-free mice after gut microbiota transplantation from db/db mice showed a significant increase in fasting blood glucose, body weight, and food and fluid intake compared with those in the controls as described previously [20]. Therefore, there is possibly a potential linkage between gut microbiota and T2DM development.

Peripheral tissues, such as skeletal muscle and liver, are considered as vital tissues in regulation of glucose metabolism [46]. In this regard, severe impairments in liver and muscle may result in the abnormality of glucose intake and usage combined with insulin resistance, which triggers the onset of diabetes [47]. Additionally, evidence has revealed that Hippo signaling is a vital regulator in the peripheral insulin pathway, maintaining glucose homeostasis by mediating the expressions of MST1, LATS1, or YAP [48]. Therefore, we detected the MST1 and LATS1 levels, and p-YAP/AYP ratio in the heart, liver, kidney, muscle, and gut tissues of db/db and pseudo germ-free mice after fecal transplantation. Negative correlations were found between fasting blood glucose and Hippo signaling. Interestingly, we observed lower Hippo signaling levels in the heart, liver, and gut in db/ db mice than those in the control group. Additionally, the host after gut microbiota transplantation from db/db mice showed decreased MST1 and LATS1 levels, and p-YAP/YAP ratio in the heart, liver, and kidney than those in m/m mice. These findings were consistent with the role of Hippo signaling in glucose metabolism via downregulation of YAP [49]. Collectively, aberrant expressions of Hippo signaling in selected peripheral tissues might contribute to the development of T2DM.

It is acknowledged that Hippo signaling is mediated by G protein coupled receptor pathway, and epinephrine or glucagon can stimulate Gs-coupled receptors, thus activating Lats1/2 kinase and inhibiting YAP function *in vitro* trials of multiple cell lines [50]. Therefore, since glucagon or epinephrine, a nonspecific activator, indirectly affects Hippo signaling, we did not observe the effects of Hippo signaling activators on glucose regulation.

Our study has several limitations. First, we should adopt more mice to minimize the difference among the groups. Second, all age groups especially the aged ones are needed. Third, tissues, including adipose tissues and various brain tissues, also should be evaluated by various techniques, including immunohistochemistry and quantitative polymerase chain reaction. Moreover, antagonists of Hippo signaling, or interference plasmid with lentivirus vectors that knockdown Hippo signaling were not conducted in this study. It is therefore further investigations are urgently needed.

To conclude, our results revealed that the aberrant expressions of Hippo signaling in selected peripheral tissues may trigger the onset of T2DM. Thus, therapeutic interventions improving Hippo signaling by gut microbiota transplantation might be beneficial to the treatment of T2DM, which provides a new insight into the study of age-related metabolic disorders in the near future.

## MATERIALS AND METHODS

### Animals

8-week-old male db/db mice (Lepr-KO/KO, *n* = 6), m/m mice (Lepr-WT/WT, *n* = 6) of the C57 BL/KS strain, and C57BL/6J male mice (*n* = 40) (Beijing Vital River Laboratory Animal Technology, Beijing, China) were housed 3-5 per cage under controlled lighting conditions (12 h light: 12 h darkness cycle), with free access to rodent feed and water. Housing conditions were in a specific pathogen–free (SPF) facility at a consistent temperature (22° C ± 2° C) combined with a relative humidity of 60% ± 5%. All experimental protocols and animal handling procedures were conducted strictly according to the recommendations in the Guide for the Care and Use of Laboratory Animals, published by the National Institutes of Health (Publications No. 80-23, revised in 1996). This study was approved by the Experimental Animal Committee of Tongji Hospital, Tongji Medical College, Huazhong University of Science and Technology (Wuhan, China).

### Measurements of metabolic parameters

During the experiments, body weight, food and fluid intake were detected via electronic scales each week, and fasting glycemia (Fasting for 8 h) was measured from a tail vein blood sample via a OneTouch Ultra blood glucose meter (LifeScan Diabetes Institute, Chesterbrook, PA, USA).

### Establishment of pseudo germ-free mouse model

C57BL/6J male mice (n = 40) weighing 20-25 g were treated with large doses of broad-spectrum antibiotics (ampicillin 1 g/L, neomycin sulfate 1 g/L, metronidazole 1 g/L; Sigma-Aldrich Co., St. Louis, MO, USA) by *ad libitum* for 2 weeks before fecal transplantation [9, 10, 20]. During the experiment, the drinking water with dissolved antibiotics was replaced every 2 days [51, 52].

### Fecal transplantation

During the establishment of pseudo germ-free mouse model, fecal samples can be prepared and stored at −80° C [9, 53]. Each mouse was respectively in a clean cage with sterilized filter paper for fecal collection [10, 20]. The feces were collected immediately into a sterilized centrifuge tube after defecation. Then, 1 g fecal samples from db/db or m/m mice were mixed with 10 ml saline solution, vortexed, and 0.2 ml of the suspension was administered to pseudo germ-free mice by oral gavage for 14 consecutive days [54].

### Western blotting

To prepare total protein extracts, selected peripheral tissues (heart, liver, kidney, right anterior foot muscle, and gut) were quickly collected after sacrifice and lysed in RIPA buffer (150 mM sodium chloride, Triton X-100, 0.5% sodium deoxycholate, 0.1% sodium dodecyl sulfate [SDS], 50 mM Tris, pH 8.0) mixed with protease and phosphatase inhibitors. The concentration of proteins in the supernatants was quantified through a BCA protein assay kit (Boster, Wuhan, China). Protein samples were denatured in loading buffer and resolved by 10% SDS-polyacrylamide gel electrophoresis, and transferred onto polyvinylidene difluoride membranes (Millipore, Bedford, MA, USA). Membranes were blocked in 5% bovine serum albumin for 1 hour before incubation with primary antibody overnight at 4° C. Membranes were washed with TBST and incubated with secondary antibodies at room temperature for 1.5 hour. The signals of proteins were then visualized by an ChemiDocXRS chemiluminescence imaging system (Bio-Rad, Hercules, CA, USA). The following antibodies were used: rabbit anti-MST1 (1:1000; Proteintech, Wuhan, China), rabbit anti-LATS1 (1:1000; Absin Bioscience Inc., Shanghai, China), rabbit anti-p-YAP (1:1000; Cell Signaling Technology, Danvers, MA, USA) and rabbit anti-YAP (1:1000; Proteintech, Wuhan, China), and horseradish peroxidase-conjugated goat anti-rabbit IgG antibody (1:5000; Affinity, Cincinnati, OH, USA).

### Statistical analysis

Data are shown as mean ± standard error of the mean. Statistical analyses were conducted using GraphPad Prism 7 (GraphPad Software, San Diego, CA, USA). Data were analyzed by 1- or 2-way analysis of variance or unpaired *t*-test. Correlation analyses were performed using Pearson’s product–moment coefficient. The *P* < 0.05 was considered statistically significant.
